# Evaluation of the relation between respiratory muscle pressure (PMUS) and diaphragmatic thickness in ICU patients ventillated with proportional assist ventillation (PAV). a preliminary study

**DOI:** 10.1186/2197-425X-3-S1-A312

**Published:** 2015-10-01

**Authors:** G Minas, N Koronakis, S Fetta, L Kypri, T Kyprianou

**Affiliations:** Nicosia General Hospital, Intensive Care Unit, Nicosia, Cyprus

## Introduction

Proportional Assist Ventillation (PAV) is a patient and user friendly mode of spontaneous mechanical ventilation which uses dynamic feedback of respiratory parameters to support the effort and assess the consequent workload overcomed by the patient´s respiratory muscles to which diaphragm contributes the most. The relationship of Pmus [1] (respiratory muscle pressure) and diaphragmatic function (displacement-excursion/ thickening ratio) has not however been adequately investigated.

## Objectives

The aim of our study is to evaluate the relation between this Pmus (cmH2O) and the diaphragmatic function as obtained by ultrasonography in ICU patients ventillated with PAV, during weaning.

## Methods

20 ICU surgical and medical patients were included in the study. All patients were off sedation able to breathe spontaneously and ventilated with PAV with a target of 6-7 ml/kg as adjusted with the gain (%support).An ultrasound study recorded the diaphragmatic displacement at the right mid axillary line and thickness at the zone of apposition [2]. The values of Ppeak, PEEP, tidal volume Vt, Sp02, compliance, % gain, displacement, Tdi (thickness at end inspiration) Tdex (thickness at end expiration) were recorded by a single operator based on the low interobserver variability [3] of the ultrasound method.

Pmus was calculated from the equation: Pmus = (Ppeak-PEEP) x ((100-gain))/gain and diaphragmatic thickening ratio from the equation: TdR = (Tdinspiration-Tdexpiration)/Tdexpiration.

Kolmogorof-Smirnof (K-S) distribution of gender, age, weight and other somatometric characteristics was normal. Data was analysed by SPSS 16.0 software using the Pearson correlation of bivariate analysis.

## Results

Data results are shown:

Pearson Correlation Pmus-TR = 0.420 **p = 0.065**

Pearson Correlation Pmus- DISPLACEM = 0.419 **p = 0.066**

## Conclusions

A positive but yet not statistically significant correlation is found both between the Pmus and the thickening ratio(TdR) of the diaphragm and the Pmus and the displacement of the diaphragmatic muscle. (Pearson correlation coefficient 0.420 p = 0.065 and 0.419 p = 0.066 respectively). The results so far indicate that the statistical significance should be evaluated with a larger number of patients and 2 observers in order to increase repeatability and reliability of the measurements.
